# Prognostic Relevance of Progesterone Receptor Levels in Early Luminal-Like HER2 Negative Breast Cancer Subtypes: A Retrospective Analysis

**DOI:** 10.3389/fonc.2022.813462

**Published:** 2022-03-28

**Authors:** Anna Diana, Francesca Carlino, Giuseppe Buono, Giuliano Antoniol, Vincenzo Famiglietti, Carmine De Angelis, Simone Carrano, Antonio Piccolo, Ferdinando De Vita, Fortunato Ciardiello, Bruno Daniele, Grazia Arpino, Michele Orditura

**Affiliations:** ^1^ Division of Medical Oncology, Department of Precision Medicine, University of Campania Luigi Vanvitelli, Naples, Italy; ^2^ Medical Oncology Unit, Ospedale del Mare, Naples, Italy; ^3^ Medical Oncology Unit, Ospedale Ave Gratia Plena, San Felice a Cancello, Caserta, Italy; ^4^ Department of Breast and Thoracic Oncology, Istituto Nazionale Tumori Istituto di Ricovero e Cura a Carattere Scientifico (IRCCS) “Fondazione G. Pascale”, Naples, Italy; ^5^ Polytechnique Montréal, Montréal, QC, Canada; ^6^ Department of Clinical Medicine and Surgery, Oncology Division, University of Naples “Federico II”, Naples, Italy

**Keywords:** breast cancer, progesterone receptor, Ki67, luminal-like subtype, prognosis

## Abstract

**Introduction:**

In luminal-like early breast cancer (BC), the lack of Progesterone Receptor (PR) expression generally correlates with more aggressive behavior but the clinical validity of low PR levels remains a debated issue.

**Methods:**

The main aim of this retrospective analysis was to assess the survival outcome (Breast cancer specific survival, BCSS) in a cohort of 687 luminal-like HER2 negative early BC patients treated at our Institutions from January 2000 to December 2018, using a sub-classification of tumors in subgroup 1 (PR high/Ki67 low), subgroup 2 (PR high/Ki67 high), subgroup 3 (PR low/Ki67 low), subgroup 4 (PR low/Ki67 high) according to PR and Ki67 values.

**Results:**

At a median follow-up of 7 years, BCSS rates were 96.3%, 89%, 86.8% and 85% in the subgroup 1, 2, 3, 4 respectively. Overall, a statistically significant difference in BCSS rates was observed among the 4 subgroups (p=0.0036). On univariate analysis, post-menopause, older age (≥ 50 years), low PR and high Ki67 expression, poorly differentiated grade and size ≥ 2 cm as well as luminal B-like tumors (subgroups 2, 3, 4) were significantly associated with a worse BCSS. Multivariate analysis identified grade, size and subgroup classification of BC as independent prognostic markers of poorer outcome. In particular, subgroups 4, 3 and 2 displayed a significantly higher risk of BC-related death (HR=4.11; p=0.008; HR=3.43; p=0-007; HR=2.57; p=0.020, respectively) when compared to subgroup 1.

**Conclusions:**

Our results support the usefulness of PR and Ki67 levels as prognostic markers, corroborating their crucial role in the decision-making process of patients with luminal-like HER2 negative early BC. Clinical application of these parameters should be assessed prospectively.

## Introduction

Breast cancer (BC) is a heterogeneous disease. Based on gene expression analysis, it has been classified in five molecular or “intrinsic” subtypes linked to different prognosis and therapeutic responsiveness (1, 2). So far, high costs and technical issues have limited the use of genomic profile tools in routine clinical practice. Therefore, therapeutic decision-making process is still commonly based on clinical and immunohistochemical (IHC) characteristics, namely tumor size and grade, nodal status, hormone receptor (HR) expression, human epidermal growth factor receptor 2 (HER2) status and Ki67 values (3). According to IHC evaluation of HR, Ki67 expression levels and HER2 status, a “surrogate” classification of estrogen receptor (ER) positive (+)/HER2 negative BC in luminal A- and luminal B-like tumors has been established and widely used in clinical practice (4). A cut off value of 20% of progesterone receptor (PR) and Ki67 has been suggested to distinguish high versus low expression levels (5, 6) and is currently used to categorize luminal BC. In detail, *Luminal A-like* tumors, characterized by high PR (i.e. ≥ 20%) and low Ki67 (i.e. <20%) levels, have an excellent prognosis and endocrine sensitivity, while *luminal B-like HER2 negative* cancers, identified by low PR (i.e. < 20%) and/or high Ki67 (i.e. ≥ 20%) values, represent an extremely heterogeneous subgroup associated with a slightly unfavorable outcome (4). In this context, in the absence of available reimbursed genomic tests for widespread clinical use, the major challenge is to identify which type of luminal B-like patient could, actually, obtain additional benefit from adjuvant chemotherapy (CT) with respect to endocrine therapy (ET) alone.

Despite the well-known role of ER expression as a prognostic factor and predictor of ET sensitivity, the clinical utility of PR measurement for risk assessment and guidance for adjuvant treatment choice has long been debated and remains less clear (5). Several studies, however, correlated low or negative PR expression with a poorer prognosis (6–9) and, in the latest ASCO/CAP guidelines, the expert panel highlighted the relevance of PR levels as a prognostic marker in BC (5).

The aim of this retrospective analysis was to investigate the prognostic role of PR expression levels in a cohort of 687 luminal-like HER2 negative BC patients, using a sub-classification of luminal B-like BC according to PR and Ki67 expression.

## Materials and Methods

### Patients and Tumor Characteristics

Clinical records of 687 consecutive patients with primary resectable, N0-1 (up to 3 axillary lymph nodes involved), invasive, luminal-like HER2 negative BC referred to the Oncology Units at “Luigi Vanvitelli” and “Federico II” teaching hospitals in Naples, Italy, between January 1, 2000, and December 31, 2018, were retrieved. Follow-up was available until February 2021. Patients affected by *in situ* or *de novo* metastatic carcinoma at the time of diagnosis, as well as patients with 4 or more axillary lymph nodes involved (N2-3) were excluded. All women were treated with tamoxifen and/or aromatase inhibitors as adjuvant endocrine therapy for five years. Chemotherapy was administered to patients with high-risk features such as large tumor size, poorly differentiated grade, high Ki67, younger age and nodal involvement. Clinicopathological parameters including histological type, grade, ER, PR, Ki67 values were measured on surgical specimens by immunohistochemistry. BC was considered to be ER+ if at least 1% of invasive malignant cells exhibited nuclear staining or immunoreactivity, while a cut-off value of 20% was used to distinguish low versus high Ki67 and PR expression levels (4, 10).

All patients were categorized into four subgroups according to PR and Ki67 values, as follows: subgroup 1 or “*Luminal-A like”* (PR high/Ki67 low), subgroup 2 or *“Luminal-B like with high Ki67”* (PR high/Ki67 high), subgroup 3 or *“Luminal-B like with low PR”* (PR low/Ki67 low), subgroup 4 or “*Luminal-B like with low PR and high Ki67”* (PR low/Ki67 high).

Medical history, type of surgery, adjuvant treatments and clinicopathological characteristics of tumors were collected. All patients were treated in accordance with national and international guidelines.

### Statistical Analysis

This study was conducted to assess the survival outcome of a cohort of non-metastatic luminal-like HER2 negative BC patients. Breast cancer specific survival (BCSS) was calculated from the date of surgery to the date of cancer-related death or last follow up. Follow-up for patients who were alive at the time of database lock was censored at the date of the last follow up. Continuous variables (e.g., ER, PR and Ki67), discrete variables (e.g., age) and categorical variables (e.g., grading, histological type) were included in the analysis. Variables were dichotomized as follows: age (<50 *vs* ≥50 years), menopausal status (premenopausal *vs* postmenopausal), Ki67 (<20% *vs* ≥20%), PR (<20% *vs* ≥20%), grade (G1-G2 *vs* G3), histological type (ductal *vs* other), lymph nodal metastases (N0 *vs* N1), tumor size (<2 cm *vs* ≥2 cm), type of surgery (mastectomy *vs* conservative surgery), adjuvant chemotherapy (yes *vs* no).

The χ^2^ test was used to assess the differences in the distribution of clinicopathological variables among the subgroups. Whenever the number of expected observations were lower than five (namely for histological subtype), we also applied the Fisher exact test. The survival analysis was carried out using the Kaplan-Meier (KM) method and the log-rank test was performed to estimate the differences among the curves, while survminer R package was mainly used for curve visualization. Cox proportional hazard regressions were applied to univariate and multivariate analyses to identify independent factors affecting BCSS. Multivariate analysis includes only those variables resulted statistically significant in univariate analysis. All statistical analyses have been performed using the open source environment R, release 4.0 (see: https://cran.r-project.org/) on a MacBook Pro. In all analyses, significance was established at a p value < 0.05.

## Results

### Patients’ Characteristics

Patients’ characteristics are summarized in **Table 1**. The study enrolled 687 women stratified as follows: 267 (39%) patients fell into subgroup 1, 264 (38%) into subgroup 2, 76 (11%) into subgroup 3 and 80 (12%) into subgroup 4. Median age was 53 years (range: 25-83 years), with 60% of patients aged 50 or over. Younger age (<50 years) and premenopausal status were more frequently recorded in groups 2 and 4. About 60-70% of the tumors were smaller than 2 cm, well or moderately differentiated (G1-2) and node negative. A significant different distribution of patients by ER status in the four subgroups was found, with a higher prevalence (86.6%) of tumors with high ER positivity (≥50%).

**Table 1:** Clinicopathological characteristics of 687 patients with luminal-like HER2-negative. BC according to different PR and Ki67 expression levels (subgroups).

	N (%)	Subgroup 1 (PR≥20%/Ki67 <20%)	Subgroup 2 (PR≥20%/Ki67≥20%)	Subgroup 3 (PR<20%/Ki67<20%)	Subgroup 4 (PR<20%/Ki67≥20%)	X2	P
Total	687	267 (39%)	264 (38%)	76 (11%)	80 (12%)		
**Age**							
<50 years	274 (40%)	91 (34.0%)	119 (45.0%)	28 (36.8%)	36 (45.0%)	7.88	0.049
≥50 years	413 (60%)	176 (66.0%)	145 (55.0%)	48 (63.2%)	44 (55.0%)		
**Menopausal status**							
Post-menopausal	406 (59.1%)	176 (65.9%)	142 (53.8%)	46 (60.5%)	42 (52.5%)	9.40	0.024
Pre-menopausal	281 (40.9%)	91 (34.1%)	122 (46.2%)	30 (39.5%)	38 (47.5%)		
<2 cm	428 (62%)	192 (71.9%)	156 (59.1%)	44 (57.9%)	36 (45.0%)	22.4	<0.001
≥2 cm	259 (38%)	75 (28.1%)	108 (40.9%)	32 (42.1%)	44 (55.0%)		
**Nodal metastases**							
No	461 (67%)	197 (73.8%)	146 (55.3%)	58 (76.3%)	60 (75.0%)	27.2	<0.001
Yes	226 (33%)	70 (26.2%)	118 (44.7%)	18 (23.7%)	20 (25.0%)		
**Grading**							
G1-G2	469 (68%)	221 (82.8%)	152 (57.6%)	68 (89.5%)	28 (35.0%)	96.5	<0.001
G3	218 (32%)	46 (17.2)	112 (42.4%)	8 (10.5%)	52 (65.0%)		
**Chemotherapy**							
Yes	367 (53%)	86 (32.2%)	181 (68.6%)	36 (47.4%)	64 (80.0%)	96.4	<0.001
No	320 (47%)	181 (67.8%)	83 (31.4%)	40 (52.6%)	16 (20.0%)		
**Radiotherapy**							
No	146 (21%)	43 (16.1%)	63 (23.9%)	18 (23.7%)	22 (27.5)	7.43	0.06
Yes	541 (79%)	224 (83.9%)	201 (76.1%)	58 (76.3%)	58 (72.5%)		
**Estrogen Receptor status**							
1-9%	20 (3%)	2 (0.7%)	2 (0.7%)	2 (2.6%)	14 (17.5%)	126.8	<0.001
10-49%	72 (10.4%)	13 (4.9%)	25 (9.5%)	8 (10.5%)	26 (32.5%)		
≥ 50%	595 (86.6%)	252 (94.4%)	237 (89.8%)	66 (86.9%)	40 (50%)		
**Histological subtype**							
Ductal	592 (86%)	229 (85.8%)	227 (86.0%)	58 (76.3%)	78 (97.5-%)	14.9	0.002
Other	95 (14%)	38 (14.2%)	37 (14.0%)	18 (23.7%)	2 (2.5%)		
**Surgery**							
Mastectomy	164 (23.9%)	53 (19.9%)	69 (26.1%)	16 (21.1%)	26 (32.5%)	6.73	0.08
Breast-conserving surgery	523 (76.1%)	214 (80.1%)	195 (73.9%)	60 (78.9%)	54 (67.5%)		

Most of larger (≥2 cm) and poorly differentiated (G3) tumors were in subgroup 4 (55% and 65%, respectively). Nodal involvement was found in 33% (n=226) of the total population, with about half of the cases (n=118) belonging to group 2. Ductal carcinoma accounted for 86% of all cases (n=592), while the remaining 24% of tumors (n=95) were found to be lobular in 88, mucinous in 5, and apocrine in 2 patients, respectively. The entire cohort received ET while CT was administered to 53% of patients (n=367), distributed as follow: 32%, 69%, 47% and 80% in subgroup 1, 2, 3, 4, respectively. In particular, in the 72% (n=264) of cases a sequential anthracycline and taxane-based regimen was prescribed.

### Survival Analysis

**Figure 1:**
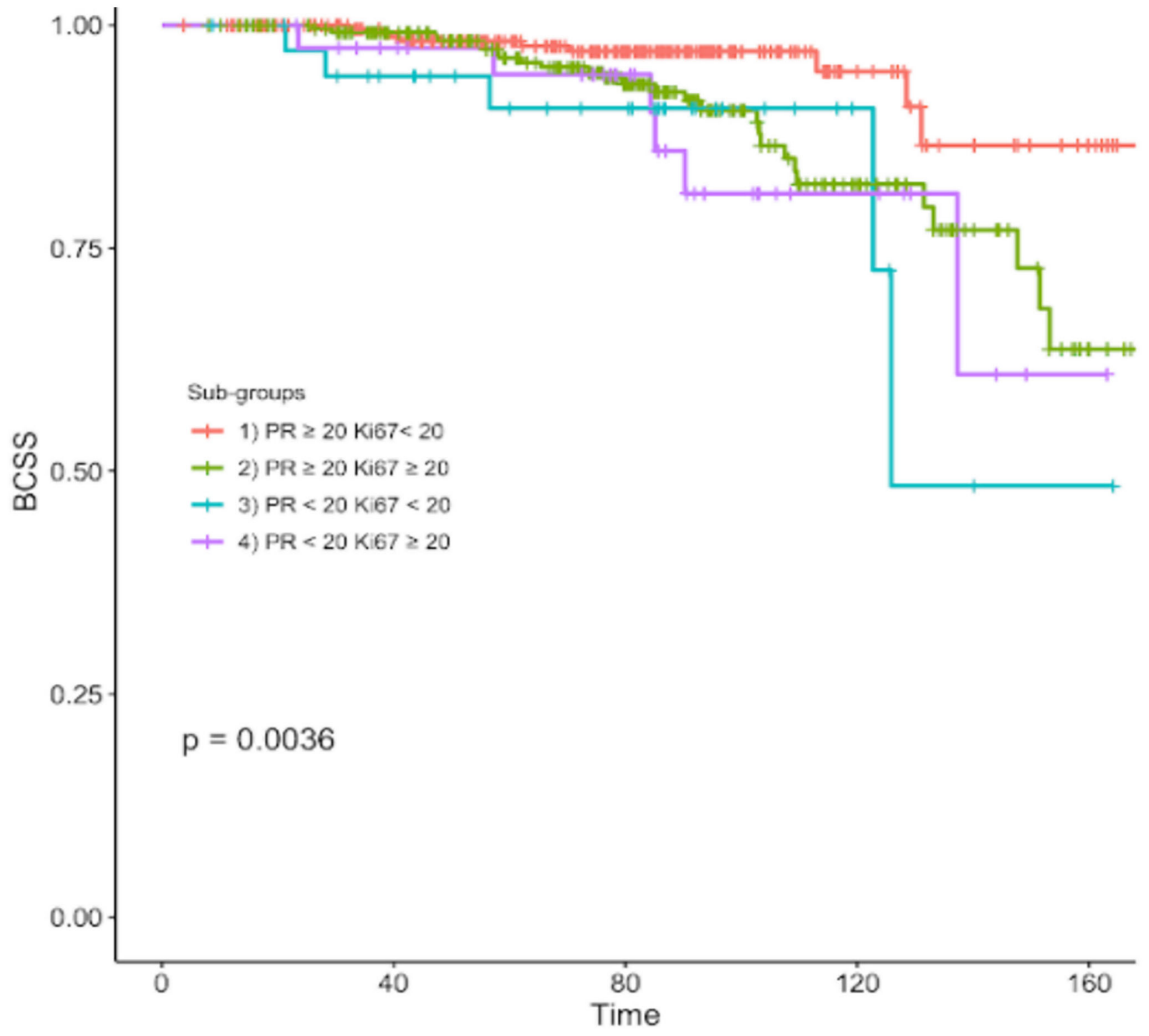
Kaplan-Meier curve of breast cancer specific survival of different subgroups according to different PR and Ki67 expression levels.

After a median follow-up of 7 years, 61 patients (9%) died of breast cancer. The median survival of all patients since diagnosis of BC was 82 months, ranging from 56 to 103 months. The resulting BCSS rates were 96.3%, 89%, 86.8% and 85% in subgroups 1, 2, 3, 4 respectively (**Table 2**). Overall, significant differences in BCSS were registered among the four subgroups (p=0.0036) (**Figure 1**).

**Table 2:** Breast cancer specific-survival rate according to different PR and Ki67 expression levels.

Sub-groups	N° of patients	N° of Deaths	BCSS rates	*p-value*
1 (PR ≥20 Ki67<20)	267	10	96,3%	0.004
2 (PR ≥20 Ki67≥20)	264	29	89,0%	
3 (PR<20 Ki67<20)	76	10	86,8%	
4 (PR<20 Ki67≥20)	80	12	85,0%	
**Total**	**687**	**61**	**91,1%**	

PR, Progesterone receptor; BCSS, Breast cancer specific-survival.

On univariate analysis, post-menopause, older age (≥ 50 years), low PR (i.e. < 10% and < 20%) and high Ki67 expression, poorly differentiated grade and size ≥ 2cm as well as the sub-classification of luminal B-like BC significantly correlated with a worse BCSS (**Table 3**).

**Table 3:** Prognostic variables for breast cancer specific survival in univariate analysis.

Variables	Univariate	
	HR (95% CI)	p-value
**Age**		
<50	1	
≥50	1.8 (1-3)	0.044
**Menopausal status**		
Pre-menopausal	1	
Post-menopausal	1.8 (1.1-3.1)	0.032
**PR**		
<20%	1	
≥20%	0.41 (0.24-0.67)	<0.001
**PR**		
<10%)	1	
≥10%	0.6 (0.3 – 0.9)	< 0.04
**Ki67**		
<20%	1	
≥20%	2.4 (1.4-3.9)	0.001
**Nodal metastases**		
No	1	
Yes	1.3 (0.79-2.2)	0.29
**Tumor size**		
<2 cm	1	
≥2 cm	2.0 (1.2-3.4)	0.007
**Grading**		
G1-G2	1	
G3	2.1 (1.3-3.6)	0.004
**Chemotherapy**		
No	1	
Yes	0.83 (0.5-1.4)	0.49
**Histological type**		
Ductal	1	
Others (lobular, mucinous, apocrine)	0.71 (0.3-1.6)	0.64
**Radiotherapy**		
No	1	
Yes	0.94 (0.53-1.7)	0.84
**Stage**		
I	1	
**II-III**	1.6 (0.94-2.8)	0.08
**Type of surgery**		
Mastectomy	1	
Breast-conserving surgery	0.69 (0.41-1.2)	0.17
**Sub-Groups**		
1 (PR ≥20 Ki67<20)	1	
2 (PR ≥20 Ki67≥20)	2.92 (1.4-0-6.26)	0.004
3 (PR<20 Ki67<20)	3.49 (2.70 – 8.63)	0.007
4 (PR<20 Ki67≥20)	4.11 (1.73-9.78)	0.001

PR, Progesterone receptor.

Multivariate analysis identified grade, size and subgroup classification of tumors as variables associated with a poorer outcome (**Table 4**). In detail, subgroups 4 (PR low/Ki67 high), 3 (PR low/Ki67low) and 2 (PR high/Ki67 high) all displayed a significantly increased risk of BC-related death (HR=4.11; p=0.008; HR=3.43; p=0-007; HR=2.57; p=0.020, respectively) when compared to subgroup 1 (PR low/Ki67 low) (**Table 4**). An additional multivariate analysis keeping Ki67 as covariate confirmed the role of PR status (<20% or ≥20%) as an independent predictor of BC survival (p=0.015). (**Table S1**).

**Table 4:** Prognostic variables for breast cancer specific-survival in multivariate analysis.

Variables	Multivariate		
	HR (95% CI)	p-value	global p-value
**Sub-Groups**			**<0.001**
1 (PR ≥20 Ki67<20)	1		
2 (PR ≥20 Ki67≥20)	2.57 (1.15 – 5.45)	0.020	
3 (PR<20 Ki67<20)	3.43 (1.38 - 8.50)	0.007	
4 (PR<20 Ki67≥20)	4.11 (1.37 - 8.17)	0.008	
**Grading**			
1-2	1		
3	1.74 (1.01 - 2.98)	0.045	
**Tumor size**			
<2 cm	1		
≥2 cm	1.66 (0.98 - 2.79)	0.054	

PR, Progesterone receptor. Bold value signify the global p-value of the statistical test performed.

## Discussion

The risk stratification process remains a critical issue in selecting the adjuvant treatment for early BC. Gene expression analysis has categorized BC into distinct molecular subtypes associated with different outcomes and treatment sensitivity (2). ER+/HER2 negative BC, representing 75-80% of all cases, include “Luminal-A like” tumors associated with good prognosis as well as ET responsiveness and “Luminal-B like” cancers, representing a heterogeneous disease with a more aggressive behavior often requiring CT (11).

The role of gene expression profiling (GEP) assays in risk stratification and treatment decisions for early BC patients is undisputed, but, unfortunately, these tests are not fully integrated in daily clinical practice due to high costs and other logistic issues. Therefore, researchers have recently re-focused on the value of traditional clinicopathological features for prognosis estimation and treatment planning, in order to assess whether IHC-based biomarkers could substitute or integrate information obtained from GEP (12, 13). ER expression represents the most important prognostic biomarker in luminal-like HER2 negative BC and the main predictor of ET responsiveness (7). Recent analyses showed that the risk of relapse and death persists despite the completion of 5 years of adjuvant ET in ER+/HER2 negative BC patients (14–16), suggesting that additional parameters, beyond ER status, should be considered to identify patients who may obtain additional benefit from CT and/or extended ET. Although 1% is the recommended cut-off to define ER positivity (5), recent evidences revealed that tumors with low ER levels (1-9%) display a clinical behavior more similar to ER-negative BC, both in terms of response to neoadjuvant chemotherapy and prognosis thus suggesting that threshold of 10% should be used in clinical practice for therapeutic decisions (17).

In this scenario, PR and Ki67 evaluation deserve a special attention. Despite the optimal threshold has not been defined yet, high Ki67 levels demonstrated to be associated with an increased risk of relapse and a worse survival (18). Furthermore, recent studies indicate that changes in Ki67 expression after neo-adjuvant ET may predict long-term outcomes, supporting its prognostic value (19). Conversely, the clinical utility of semi-quantitative assessment of PR levels is still debated and not fully elucidated. Previous exploratory analyses of multiple independent datasets have demonstrated that quantitative scoring of PR positive tumor cells (but not ER positive tumor cells) might predict BC outcome when an empiric cut-off of more than 20% for PR percentage to discriminate luminal A and luminal B-like was chosen (20). Additional data confirmed that tumors with low (i.e. <20%) or negative PR expression display a more aggressive phenotype, although its prognostic value seems to decrease after long-term follow-up (21–24).

Results from gene expression studies revealed a specific molecular profile of single HR+ BC associated with poor prognostic factors and response to endocrine therapy as well as less favorable clinical behaviour compared to double HR+ cancers (25). In particular, PAM 50 analysis showed that ER-/PR+ tumors, accounting less than 1% of all BC, are mostly basal like (50-60%), a molecular subtype generally observed in TNBC which can be easily detectable through immunohistochemistry-based method (TFF1, CK5, and EGFR positivity) (26).

Moreover, at immunohistochemical level too, ER-/PR+ BC compared to ER+/PR+ cases are more likely associated to biomarkers predicting worse prognosis such as p53 and basal cytokeratin expression, high Ki67 and MKI67 mRNA levels, as well as low E-cadherin and absence of androgen receptor (27).

Otherwise, PR represents a molecular rheostat controlling ERα transcriptional activity and regulates chromatin binding events, resulting in a unique gene expression signature associated with good prognosis (28). Therefore, the absence of PR leads to the activation of genes related to aggressive features, including myc, cyclin D1, and insulin-like growth factor receptor 1 (29, 30). Unfortunately, in our series, the number of patients with PR negative breast tumors is too small (7% of all population) to provide meaningful results.

With regard to the predictive value of PR levels, clinical studies reported controversial results (31, 32). Some authors concluded that patients affected by PR negative tumors, unlikely to obtain benefit from adjuvant ET, could gain a survival advantage from adjuvant CT (21, 23). Moreover, a retrospective analysis from three adjuvant clinical trials supported this hypothesis showing that low PR expression could be predictive of additional benefit from CT compared to ET alone, regardless of ER positivity (33). A large metanalysis including 20 trials and more than 20,000 patients with ER+ early BC revealed that tamoxifen improves relapse-free survival regardless of PR status, age, nodal status, or use of adjuvant CT (7). Similarly, a retrospective analysis from the ATAC and BIG 1-98 trials showed that PR expression did not affect the survival advantage obtained from tamoxifen or an aromatase inhibitor, but confirmed a significant role for outcome prediction in both treatment arms (33, 34). Overall, these trials suggest that PR expression has an intrinsic prognostic effect, although its predictive relevance remains controversial (35).

Additionally, a large amount of data reported that IHC PR expression levels correlate with the Oncotype DX recurrence score (RS) and support the combined use of PR and mitotic rate as a surrogate marker for Oncotype RS (36–38).

Based on these observations, expert panels agreed that low PR expression can be used as a prognostic determinant for Luminal-like tumors (4), recommending its use in combination with others pathological factors such as Ki67, histological grade and tumor stage (4, 6).

In order to get a deeper insight into the prognostic significance of PR, we retrospectively analyzed a cohort of 687 non-metastatic N0-1 luminal-like HER2 negative BC patients stratified into four subgroups based on PR and Ki67 expression levels as measured by IHC, with a cut-off of 20% according with the 2013 Saint Gallen International Breast Cancer Conference (4). The survival analysis showed a statistically significant difference in terms of BCSS among the four subgroups. As expected, the luminal-B like subtype displayed a more aggressive clinical behavior and an unfavorable prognosis when compared to luminal-A like cancers. Moreover, when looking at the survival curves, each subclass of luminal-B like BC patients presented a significantly different risk of death. Of note patients belonging to subgroup 4 (PR-low/Ki67-high) were more commonly younger and premenopausal women affected by tumor with more aggressive features (large size, poorly differentiated grade). All these unfavorable characteristics could have contributed to the worst prognosis of these patients, for whom a 4-fold increased risk of cancer-related death was reported compared with patients with Luminal-A like BC, followed by subgroup 3 (PR<20% and Ki67<20%), and 2 (PR≥20% and Ki67≥20%). Looking at the hazard ratio of each subgroup of luminal B-like tumors, patients with low PR (subgroup 3) had a higher risk of BC mortality than those with high Ki67 (subgroup 2) (HR= 3.43 and HR= 2.57, respectively). In addition, to further explore the prognostic role of PR levels, we performed an additional multivariate analysis keeping Ki67 as covariate which confirmed PR status as a powerful and independent predictor of BC survival.

Our study and findings have strengths and limitations. Our cohort of patients is fully characterized with regard to clinical and tumor features and was evaluated and treated in two teaching hospitals, which ensure high quality pathological evaluation and medical treatments in line with the best international standards. Furthermore, in the present study, the sub-classification of ER+/HER2 negative BC according to PR and Ki67 levels proved its prognostic significance despite the majority of cases exhibited strong ER positivity (only 3% of patients with ER< 10%). Our results underline that additional factors, beyond the ER status, should be collectively considered to provide a reliable prediction of survival outcomes assisting physicians’ treatment decisions process.

Our study has several limitations. First, the possibility of bias with respect to different chemotherapy regimens adopted over the course of 18 years cannot be completely ruled out due to the retrospective nature of our study. However, we performed an additional exploratory analysis showing no statically significant differences in patients survival outcome according to the various adjuvant chemotherapy regimens used (data not shown).

In addition, it should be noted that during this observation period, several methods for hormone receptor testing have been developed and applied, while specific guidance on the best antibody, assay, and scoring system to improve reproducibility and reduce interobserver variation is still lacking. In our study, although a comprehensive immunohistochemical re-evaluation of PR expression was not performed, the Ventana 1E2 clone PR IHC assay was found to be widely used. However, a potential limitation of the present analysis could be represented by the variability in the preanalytical procedures and immunohistochemical evaluation of PR status over the years and between the two different academic laboratories.

Our findings, according to previous retrospective analyses and metanalyses (28, 33, 39, 40, 41), suggest a prognostic role for PR expression levels in luminal-like HER2 negative BC that should be confirmed prospectively.

## Conclusions

In conclusion, despite the limitations due to the retrospective nature of the study, our findings support the importance of IHC-based evaluation of PR expression levels combined with Ki67 status to sub-classify, among patients with Luminal-B like BC, groups with different prognosis, which could be useful in modulating and personalizing BC adjuvant treatments. Therefore, semi-quantitative measurement of PR and Ki67 expression levels maintains clinical relevance for risk estimation and treatment guidance even in the era of genomic profiling.

## Data Availability Statement

The raw data supporting the conclusions of this article will be made available by the authors, without undue reservation.

## Ethics Statement

The studies involving human participants were reviewed and approved by Ethical Committee of the University of Campania “Luigi Vanvitelli” (Naples, Italy). The patients/participants provided their written informed consent to participate in this study.

## Author Contributions

Conceptualization: AD, FCa, and MO. Methodology: GAn, VF, GB, and AD. Resources: CDA, SC, and AP. Data curation: GAn, VF, AD, FDV, FCa. Writing—original draft preparation: AD and FCa. Writing—review and editing: MO, AD, FCa and GB. Supervision: CDA, GrA, BD. Validation: CDA, FDV, and FCi, BD, GAr, and MO. All authors have read and agreed to the published version of the manuscript.

## Conflict of Interest

The authors declare that the research was conducted in the absence of any commercial or financial relationships that could be construed as a potential conflict of interest.

## Publisher’s Note

All claims expressed in this article are solely those of the authors and do not necessarily represent those of their affiliated organizations, or those of the publisher, the editors and the reviewers. Any product that may be evaluated in this article, or claim that may be made by its manufacturer, is not guaranteed or endorsed by the publisher.

## Supplementary Material

Supplementary Table 1 Prognostic variables for breast cancer specific-survival in multivariate analysis keeping Ki67 as covariate.
