# Supplementary material for: Prognostic Relevance of Progesterone Receptor Levels in Early Luminal-Like HER2 Negative Breast Cancer Subtypes: A Retrospective Analysis
Source: Front Oncol. 2022 Mar 28;12:813462. doi: 10.3389/fonc.2022.813462 (PMC8996175; doi:10.3389/fonc.2022.813462)
Supplement: Supplementary Table 1 — Prognostic variables for breast cancer specific-survival in multivariate analysis keeping Ki67 as covariate. [file Table_1.docx]

| **Variables** | **Multivariate** | | |
| --- | --- | --- | --- |
|  | **HR (95% CI)** | **p-value** | **global p-value (Likelihood test)** |
| **PR** | | | **<0.0001** |
| <20% | 1 | 0.015 |  |
| ≥20% | 0.52 (0.30-0.882) |  |  |
| **Ki67** | | |  |
|  | 5.60 (1.17-26.76) | 0.03 |  |
| **Age** | | |  |
|  | 1.03 (1.00-1.056) | 0.006 |  |
| **Grading** | | |  |
| 1-2 | 1 | 0.09 |  |
| 3 | 1.62 (0.91-2.86) |  |  |
| **Tumor size** | | |  |
| <2 cm | 1 | 0.062 |  |
| ≥2 cm | 1.64 (0.97-2.76) |  |  |

**Table S1. Prognostic variables for breast cancer specific-survival in multivariate analysis keeping Ki67 as covariate.**
